# Molecular characteristics of *Clostridium difficile* in children with acute gastroenteritis from Zhejiang

**DOI:** 10.1186/s12879-020-05030-6

**Published:** 2020-05-13

**Authors:** Huiqun Shuai, Qiao Bian, Yun Luo, Xiaohong Zhou, Xiaojun Song, Julian Ye, Qinghong Huang, Zhaoyang Peng, Jun Wu, Jianmin Jiang, Dazhi Jin

**Affiliations:** 1 Xiacheng District Center for Disease Control and Prevention, Hangzhou, China; 2 School of Medicine, Ningbo University, Ningbo, China; 3 Department of Microbiology, Zhejiang Provincial Center for Disease Control and Prevention, Hangzhou, China; 4 School of Biotechnology and Biomolecular Sciences, University of New South Wales, Sydney, Australia; 5 Centre of Laboratory Medicine, Zhejiang Provincial People Hospital, People’s Hospital of Hangzhou Medical College, Hangzhou, China; 6 Department of Clinical Laboratory, The Children’s Hospital of Zhejiang University School of Medicine, Hangzhou, China; 7 National Clinical Research Center for Child Health, Hangzhou, China; 8 Lin’an District Center for Disease Control and Prevention, Hangzhou, China; 9 Key Laboratory of Vaccine, Prevention and Control of Infectious Disease of Zhejiang Province, Hangzhou, China; 10 School of Laboratory Medicine, Hangzhou Medical College, No. 481, Binwen Road, Hangzhou Zhejiang, China

**Keywords:** Molecular characteristics, *Clostridium difficile*, Children, Acute gastroenteritis

## Abstract

**Background:**

*Clostridium difficile* infection (CDI) has an increasing pediatric prevalence worldwide. However, molecular characteristics of *C. difficile* in Chinese children with acute gastroenteritis have not been reported.

**Methods:**

A five-year cross-sectional study was conducted in a tertiary children’s hospital in Zhejiang. Consecutive stool specimens from outpatient children with acute gastroenteritis were cultured for *C. difficile*, and isolates then were analyzed for toxin genes, multi-locus sequence type and antimicrobial resistance. Diarrhea-related viruses were detected, and demographic data were collected.

**Results:**

A total of 115 CDI cases (14.3%), and 69 co-infected cases with both viruses and toxigenic *C. difficile,* were found in the 804 stool samples. The 186 *C. difficile* isolates included 6 of toxin A-positive/toxin B-positive/binary toxin-positive (A^+^B^+^CDT^+^), 139 of A^+^B^+^CDT^−^, 3 of A^−^B^+^CDT^+^, 36 of A^−^B^+^CDT^−^ and 2 of A^−^B^−^CDT^−^. Sequence types 26 (17.7%), 35 (11.3%), 39 (12.4%), 54 (16.7%), and 152 (11.3%) were major genotypes with significant differences among different antimicrobial resistances (Fisher's exact test, *P* < 0.001). The A^−^B^+^ isolates had significantly higher resistance, compared to erythromycin, rifampin, moxifloxacin, and gatifloxacin, than that of the A^+^B^+^ (*χ*^*2*^ = 7.78 to 29.26, *P* < 0.01). The positive CDI rate in infants (16.2%) was significantly higher than that of children over 1 year old (10.8%) (*χ*^*2*^ = 4.39, *P* = 0.036).

**Conclusions:**

CDI has been revealed as a major cause of acute gastroenteritis in children with various genotypes. The role of toxigenic *C. difficile* and risk factors of CDI should be emphatically considered in subsequent diarrhea surveillance in children from China.

## Background

*Clostridium difficile* is a Gram-positive, anaerobic, spore forming bacterium that leads to healthcare-associated diarrhea and can be as life-threatening as pseudomembranous colitis, toxic megacolon, intestinal perforation, and septic shock [1]. It has been reported that in 2011 *C. difficile* was responsible for almost half a million infections and was associated with approximately 29,000 deaths in the United States [2]. The estimated incidence of community-associated *Clostridium difficile* infection (CA-CDI) and health care–associated CDI in ages one and seventeen was 17.9 and 6.3 per 100, 000, respectively [2].

The rate of pediatric CDI-related hospitalizations has increased in the past decade in North America and Europe [3–6]. In the USA, the national rates of CDI-related pediatric hospitalizations have increased from 7.24 to 12.80 per 10,000 hospital admissions, in more than 3700 hospitals, between 1997 and 2006 [3]. One retrospective analysis revealed a 53% increase in the annual incidence density from 2001 to 2006, of 2.6 to 4.0 cases per 1000 admissions involved with 4895 CDI children, from 22 tertiary-care pediatric hospitals [4]. The CDI incidence was 6.6 cases/1000 admissions in a large pediatric hospital in Italy, where most symptomatic children less than 3 years old only had positive *C. difficile* culture without other gastrointestinal pathogens [5]. CDI has been reported in Asian children with inflammatory bowel disease, cancer and acute gastroenteritis [7–9]. However, data on *C. difficile* in children from China, including CDI rates, CDI related hospitalizations etc., were scarce. CDI was more difficult to identify in children than in adults, due to *Clostridium difficile* colonization and co-infections with various viruses (especially norovirus and caliciviruses) [9–11]. A literature review found it hard to draw any meaningful conclusions given the diversity of studies regarding the detection time, methods, the organisms tested for and the lack of cases definition of CDI in children under 5 years [12]. Testing for CDI should be routinely standardized according to new clinical practice guidelines [13]. The *C. difficile* tests in infants under 1 year were usually not recommended in the USA and UK [13, 14]. However, one retrospective cohort study found that 26% of children hospitalized with CDIs were infants and 5% were neonates [4]. The identification of CDI in pediatric population was quite complicated.

Acute gastroenteritis is still a serious public health problem in China, with more than 10,000 children dying from diarrhea annually [15]. Rotavirus group A, norovirus, *Shigella* spp., diarrheagenic *Escherichia coli* and *Salmonella* spp. are the most frequent pathogens in acute diarrhea in children [15]. Previous studies on CDI in China mostly focused on adult patients with hospitalizations [16], or specific conditions such as cancer [17], hematological malignancies [18], pregnancy [19] and advanced age [20]. Limited information is available regarding pediatric CDI and the molecular characteristics of *C. difficile* in children from China.

Although *C. difficile* colonization has been reported in northern Chinese infants [21, 22], *C. difficile* in children with acute gastroenteritis has not yet been studied, and CA-CDI in children was not mentioned in China. We conducted a five-year cross-sectional study on outpatient children with acute gastroenteritis, in a tertiary children’s hospital from eastern China, and investigated the molecular characteristics of *C. difficile,* including toxin genes, genotypes and antimicrobial susceptibility. Our study firstly presented the data on CA-CDI in children with acute gastroenteritis in Zhejiang, China, and also provided the pilot evidence to further study clinical significance of routine testing *C. difficile* in children.

## Methods

### Study design

This cross-sectional study was conducted in the Outpatient and Emergency departments of the Children’s Hospital of Zhejiang University School of Medicine, from February 2013 to December 2017, excluding October to December in 2013 and 2014. This tertiary hospital is the largest comprehensive center for pediatric health care in Zhejiang Province. Clinical stool samples were collected from selected outpatient children with acute diarrhea during the study period and then transported to the Xiacheng District Center for Disease Control and Prevention (XCCDC), within 24 h. Each sample was divided into two aliquots (1 mL/each) for further testing. This study was approved by the institutional review boards of the XCCDC. The informed consent requirement was waived due to no more than minimal risk involved in this study.

### Data collection and viral detection

According to the guidelines of the Society for Healthcare Epidemiology of America and the Infectious Diseases Society of America (SHEA/IDSA) [23], inclusion criteria were present as follows. Outpatients who suffered from acute diarrhea with more than 3 fluid, loose, or unformed stools within 24 h were sampled for this study, and all patients belonged to CA-CDI described as below. CA-CDI was defined by the presence of diarrhea symptoms and a positive test for toxigenic *C. difficile*, of which the onset of diarrhea occurred in the community or within 48 h after hospital admission, and had not been hospitalized within the previous 12 weeks [24]. Exclusion criteria were outpatients over 18 years old, patients with diarrhea who have been admitted over 48 h, and patients with underlying conditions (malignancy, immunodeficiency, abdominal surgery, hematological disease and hematopoietic stem cell transplantation). Duplicated stool samples from the same patients were removed. Clinical information on age, gender, school attendance and presenting symptoms, including fever and diarrhea, were collected.

Viral nucleic acids (DNA or RNA) were extracted from each stool sample using the appropriate kits (QIAgen, Inc., Valencia, CA, USA). The fluorescent real time PCR assays for rotavirus group A and B, norovirus genogroup I and II, astrovirus, sapovirus, adenovirus, and other viruses were used according to the manufacturer’s instructions (Shanghai ZJ Bio-Tech Co., Ltd., Shanghai, China).

### *C. difficile* culture

For *C. difficile* culture, cefoxitin-cycloserine fructose agar (CCFA, Oxoid, UK), supplemented with 7% sterile defibrinated sheep blood, was used for selective isolation. Stool samples were primarily treated with purified ethanol and plated onto a CCFA medium (as described previously) [25]. After anaerobic culture at 37 °C for 48 h (GENbag anaer, bioMérieux, France), all colonies were identified according to special odor, characteristic morphology, and gram staining, as previously reported [26]. All isolates were stored at − 80 °C in brain-heart infusion broth, supplemented with 10% glycerol, for subsequent analysis [25].

### DNA extraction and PCR of *C. difficile* toxin genes

*C. difficile* isolates were recovered on blood agar plates and extracted for genomic DNA with the QIAamp DNA blood Mini Kit (Valencia, CA, USA), according to the manufacturer’s instructions. The housekeeping gene *tpi*, toxin genes A and B (*tcdA*, *tcdB*) and binary toxin genes A and B (*cdtA*, *cdtB*) were amplified as previously reported [27–29]. The PCR product of the *tpi* gene was 230 bp in *C. difficile* isolates. The length of the *tcdA* gene was 369 bp for toxin A^+^B^+^ strains, and 110 bp for toxin A^−^B^+^ strains. The length of the *tcdB*, *cdtA*, and *cdtB* genes was 688 bp, 375 bp, and 478 bp, respectively. *C. difficile* standard strains, including BAA-1803 and BAA-1870, were used as positive controls for *tcdA* and *tcdB* and the binary toxin genes. With BAA-1801 and ATCC-700057 as negative controls for all the toxin genes (American Type Culture Collection, Manassas, VA, USA). The positive, negative, and blank controls were examined in each experiment as parallel.

### Multi-locus sequence typing (MLST)

MLST was performed as previously reported [28]. Seven housekeeping loci (*adk*, *atpA*, *dxr*, *glyA*, *recA*, *sodA*, and *tpi*) were amplified using PCR. The PCR products were identified with a 3730 XL DNA analyzer (Applied Biosystems). Data for *C. difficile* alleles and sequence types (STs) were submitted to the public MLST online database (https://pubmlst.org/cdifficile/).

### Antimicrobial susceptibility test and drug-resistant genes

A total of 12 antibiotics, including fusidic acid, ciprofloxacin, piperacillin-tazobactam (PIP-TAZ), metronidazole, rifampin, moxifloxacin, gatifloxacin, vancomycin, clindamycin, levofloxacin, tetracycline, and erythromycin were tested with the agar dilution method, according to the CLSI guideline (M11-A8, [30]). The breakpoints were determined according to the previous study [25]. Intermediate resistance was regarded as non-susceptible in later analysis. Multi-drug resistance (MDR) was defined as resistance to at least three classes of antibiotics [31]. *Bacteroides fragilis* (ATCC 25285) and *C. difficile* (ATCC 700057) were included in each run for quality control. The erythromycin- and clindamycin-resistant isolates were tested for the presence of the *ermB* gene. The tetracycline-resistant isolates were tested for the presence of the *tetM* gene, both according to previous publications [32, 33].

### Data analysis

Data was analyzed with Statistical Package for Social Sciences (SPSS, Chicago, IL, USA), version 25.0 and Microsoft Excel. The *χ*^*2*^ test, or Fisher's exact Test, was used to analyze correlations among STs, toxin gene profiles and antimicrobial susceptibility patterns of *C. difficile* strains. A *P* value of < 0.05 was considered statistically significant.

## Results

### Collection of *C. difficile* isolates

**Fig. 1:**
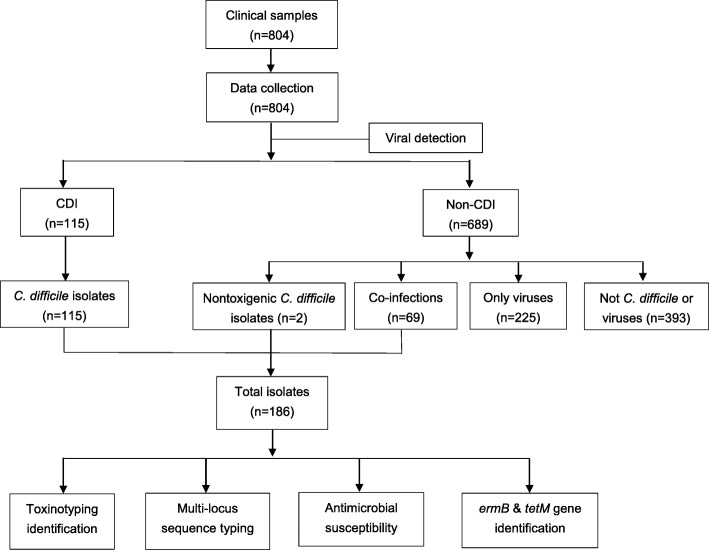
Flow diagram of data collected during this study (February 2013 to December 2017)

A total of 804 outpatient children were enrolled in this cross-sectional study, from 2013 to 2017. Stool samples were collected from each outpatient and analyzed as designed (Fig. 1). The demographic information is shown in Table 1. The overall median and interquartile range (IQR) of age was 0.67 (0.38–1.00) year old. The number of outpatients grouped by age of under six months, six months to one year, one to two years and over two years were 259, 266, 162 and 117, respectively. Moreover, 777 (96.6%) of the outpatients were scattered children and 141 (17.5%) had a fever (> 38.5 °C). In total, 115 (14.3%) cases were identified as CA-CDI, 69 (8.6%) cases had co-infections with viruses and 393 (48.9%) cases had neither *C. difficile* nor virus infections. A total of 294 (36.6%) outpatients identified positively for viruses, shown in Table 1.

**Table 1:** Clinical information of outpatients participated in this study

Characteristics	2013 (*n* = 84)	2014 (*n* = 102)	2015 (*n* = 223)	2016 (*n* = 203)	2017 (*n* = 192)	Total (*n* = 804)
Gender, male n, (%)	55 (65.5)	70 (68.6)	128 (57.4)	129 (63.5)	119 (62.0)	501 (62.3)
Age (yr), *Median*, (*IQR*)	0.50 (0.25, 0.73)	0.50 (0.25, 1.00)	0.75 (0.42, 1.25)	0.75 (0.33, 1.25)	0.79 (0.47, 1.00)	0.67 (0.38, 1.00)
Age (yr) n, (%)						
< 6 months (ms)	40 (47.6)	36 (35.3)	70 (31.4)	65 (32.0)	48 (25.0)	259 (32.2)
6 ms~	32 (38.1)	40 (39.2)	67 (30.0)	67 (33.0)	60 (31.3)	266 (33.1)
1 yr~	6 (7.1)	16 (15.7)	43 (19.3)	42 (20.7)	55 (28.6)	162 (20.1)
2 yr~	6 (7.1)	10 (9.8)	43 (19.3)	29 (14.3)	29 (15.1)	117 (14.6)
Occupation, Scattered children	81 (96.4)	101 (99.0)	216 (96.9)	193 (95.1)	186 (96.9)	777 (96.6)
Fever, > 38.5 °C	17 (20.2)	24 (23.5)	66 (29.6)	44 (21.7)	65 (33.9)	216 (26.9)
*C. difficile* isolates n, (%)	11 (13.1)	35 (34.3)	60 (26.9)	36 (17.7)	44 (22.9)	186 (23.1)
Only toxigenic *C. difficile*, CA-CDI	10 (11.9)	23 (22.5)	40 (17.9)	26 (12.8)	16 (8.3)	115 (14.3)
Co-infections	1 (1.2)	11 (10.8)	19 (8.5)	10 (4.9)	28 (14.6)	69 (8.6)
Total viral infections	4 (4.8)	23 (22.5)	88 (39.5)	63 (31.0)	116 (60.4)	294 (36.6)
Rotavirus group A^a^	2 (2.4)	0	44 (19.7)	30 (14.8)	47 (24.5)	123 (15.3)
Norovirus GI & GII^b^	1 (1.2)	20 (19.6)	19 (8.5)	26 (12.8)	32 (16.7)	98 (12.2)
Astrovirus	1 (1.2)	0	3 (1.3)	0	3 (1.6)	7 (0.9)
Sapovirus	0	1 (1.0)	9 (4.0)	3 (1.5)	3 (1.6)	16 (2.0)
Adenovirus	0	2 (2.0)	4 (1.8)	3 (1.5)	1 (0.5)	10 (1.2)
Multiple viruses	0	0	9 (4.0)	1 (0.5)	30 (15.6)	40 (5.0)

a: No case was positive for Rotavirus group B;

b: Three cases were positive for Norovirus GI type, one in 2013, two in 2017 including one co-infected with Rotavirus group A

### Toxin genes and MLST for *C. difficile* isolates

A total of 186 *C. difficile* isolates, including 6 A^+^B^+^CDT^+^, 139 A^+^B^+^CDT^−^, 3 A^−^B^+^CDT^+^, 36 A^−^B^+^CDT^−^ and 2 A^−^B^−^CDT^−^, were obtained from 804 stool samples. Totally, 27 STs were identified, with 7 new STs (ST513, 515, 526, 627, 628, 629, and 630), and none of the hypervirulent, epidemic ST1 (ribotype 027) isolates were detected in this study. The most prevalent type was ST26 (*n* = 33, 17.7%), followed by ST54 (*n* = 31, 16.7%), ST39 (*n* = 23, 12.4%), ST35 (*n* = 21, 11.3%), and ST152 (n = 21, 11.3%). ST83 and ST627 were found to be non-toxigenic (A^−^B^−^CDT^−^). All ST37, 39, 81, and 630 isolates were A^−^B^+^, with the remaining ST types being A^+^B^+^. A total of nine CDT^+^ strains included 3 of ST39, 3 of ST54, 2 of ST152 and 1 of ST15. The distribution of STs was relatively different in outpatient children younger and older than 1 year. ST26 (*n* = 29, 24.0%), ST54 (n = 21, 17.4%) and ST35 (*n* = 13, 10.7%) were the major STs in infants, whereas ST39 (*n* = 11, 17.5%), ST54 (*n* = 10, 15.9%) and ST152 (n = 10, 15.9%) were the predominant STs in children over 1 year old.

### Antimicrobial susceptibility and drug-resistant genes

The antimicrobial susceptibility patterns of all the 186 *C. difficile* isolates are summarized in Table 2. All isolates were susceptible to metronidazole, vancomycin and PIP-TAZ, and resistant to ciprofloxacin. Resistance rates varied for other antimicrobials. The resistance rates of rifampin, moxifloxacin, gatifloxacin and tetracycline were 3.8, 7.5, 7.5 and 9.1%, respectively. However, the resistance rates to clindamycin, erythromycin, fusidic acid and levofloxacin were 85.5, 86.0, 69.4 and 79.6%, respectively. The 131 isolates were intermediate to levofloxacin, while only 17 isolates were resistant to it. A high resistance rate to MDR (89.2%, 166/186) was observed in these isolates. As for two non-toxigenic isolates, one was resistant to erythromycin and ciprofloxacin, intermediate to levofloxacin, and susceptible to other nine antimicrobials, and the other was resistant to ciprofloxacin, and susceptible to all eleven antimicrobial agents. The *ermB* gene was detected in 83.9% (141/168) of the erythromycin- and clindamycin-resistant isolates, while the *tetM* gene was present in 88.2% (15/17) of the tetracycline-resistant isolates.

**Table 2:** Correlations among MLST types, toxin genotypes, and antimicrobial susceptibility patterns of the 186 *C. difficile* isolates

Antimicrobial agent	Total no. (%) of all the isolates (*n* = 186)	MLST types (no. [%] of non-susceptible isolates)						Analysis results^b^		Toxinotypes^c^ (no. [%] of non-susceptible isolates)		Analysis results	
		ST26 (*n* = 33)	ST35 (*n* = 21)	ST39 (*n* = 23)	ST54 (*n* = 31)	ST152 (*n* = 21)	Other STs^a^ (*n* = 57)	*χ*^*2*^	*P* value	A^+^B^+^ (*n* = 145)	A^−^B^+^ (*n* = 39)	*χ*^*2*^	*P* value
Clindamycin	159 (85.5)	33 (100.0)	21 (100.0)	18 (78.3)	30 (96.8)	13 (61.9)	44 (77.2)	F	< 0.001	125 (86.2)	34 (87.2)	0.02	0.875
Erythromycin	160 (86.0)	33 (100.0)	21 (100.0)	23 (100.0)	30 (96.8)	7 (33.3)	46 (80.7)	F	< 0.001	120 (82.8)	39 (100.0)	7.78	0.005
Fusidic acid	129 (69.4)	12 (36.4)	15 (71.4)	15 (65.2)	28 (90.3)	20 (95.2)	39 (68.4)	30.18	< 0.001	102 (70.3)	27 (69.2)	0.02	0.893
Rifampin	7 (3.8)	0	0	5 (21.7)	0	1 (4.8)	1 (1.8)	F	0.001	2 (1.4)	5 (12.8)	8.09	0.004
Levofloxacin	148 (79.6)	19 (57.6)	21 (100.0)	16 (69.6)	24 (77.4)	21 (100.0)	47 (82.5)	F	< 0.001	117 (80.7)	30 (76.9)	0.27	0.602
Moxifloxacin	14 (7.5)	0	0	6 (26.1)	0	1 (4.8)	7 (12.3)	F	0.001	3 (2.1)	11 (28.2)	29.26	< 0.001
Gatifloxacin	14 (7.5)	0	0	6 (26.1)	0	1 (4.8)	7 (12.3)	F	0.001	3 (2.1)	11 (28.2)	29.26	< 0.001
Tetracycline	17 (9.1)	0	11 (52.4)	0	1 (3.2)	0	5 (8.8)	F	< 0.001	13 (9.0)	4 (10.3)	0.06	0.805
Metronidazole	0	0	0	0	0	0	0	N/A	N/A	0	0	N/A	N/A
Vancomycin	0	0	0	0	0	0	0	N/A	N/A	0	0	N/A	N/A
PIP-TAZ	0	0	0	0	0	0	0	N/A	N/A	0	0	N/A	N/A
Ciprofloxacin	184 (100.0)	33 (100.0)	21 (100.0)	23 (100.0)	31 (100.0)	21 (100.0)	57 (100.0)	N/A	N/A	145 (100.0)	39 (100.0)	N/A	N/A
MDR	166 (89.2)	33 (100.0)	21 (100.0)	21 (91.3)	30 (96.8)	15 (71.4)	46 (80.7)	F	0.001	129 (89.0)	37 (94.9)	0.64	0.425

^a^: Of two non-toxigenic isolates, one (50.0%) was non-susceptible to erythromycin and levofloxacin; both of them (100.0%) were non-susceptible to ciprofloxacin and susceptible to other antimicrobial agents. None of non-toxigenic MDR isolates were found;

^b^: F: Fisher's exact test; N/A: data not applicable;

^c^: Non-toxigenic (A^−^B^−^) isolates were not included

The correlations between genotypes and antimicrobial susceptibility patterns are shown in Table 2. The antimicrobial patterns among major STs differed significantly (Fisher's exact test or *χ*^*2*^ = 36.09, *P* < 0.001) as below. In comparison with other STs, ST26 isolates had low resistance rates to fusidic acid and levofloxacin, ST35 isolates had a high resistance rate to tetracycline, and ST39 isolates had high resistance rates to rifampin, moxifloxacin, and gatifloxacin. Additionally, ST152 isolates exhibited comparatively lower resistance to clindamycin and erythromycin than the other major STs. The clindamycin resistance rate of the two non-toxigenic isolates was lower than that of toxigenic isolates (Fisher's exact test, *P* = 0.020). Furthermore, the resistance rates of erythromycin, rifampin, moxifloxacin, and gatifloxacin in A^−^B^+^ isolates were higher than those in the A^+^B^+^ isolates (*χ*^*2*^ = 7.78–29.26, *P* < 0.005).

### Epidemiology

A total of 294 (36.6%) stools tested positive for viral infections, of which 123 (41.8%) were positive for rotavirus group A, 2 (0.7%) for norovirus genotype GI and 96 (32.7%) for GII, 7 (2.4%) for astrovirus, 16 (5.4%) for sapovirus, 10 (3.4%) for adenovirus, and 40 (13.6%) for multiple virus infections. None of them tested positive for rotavirus group B. The positive rate for viruses was much higher in children older than 1 year (59.9%, 167/279) than in infants (24.2%, 127/525) (*χ*^*2*^ = 99.91, *P* < 0.001).

For the 69 (8.6%) pediatric outpatients co-infected with both viruses and toxigenic *C. difficile*, 17 (24.6%) were co-infected with rotavirus, 30 (43.5%) with norovirus, 1 (1.4%) with astrovirus, 7 (10.1%) with sapovirus, 2 (2.9%) with adenovirus and 12 (17.4%) were co-infected with multiple viruses, including rotavirus and one or more other viruses. The co-infection rate was significantly higher in children older than 1 year (11.8%, 33/279) than in infants (6.9%, 36/525) (*χ*^*2*^ = 5.74, *P* = 0.017). However, the positive rate of CA-CDI in infants (16.2%, 85/525) was significantly higher than that in children older than 1 year (10.8%, 30/279), in the 115 CA-CDI cases (*χ*^*2*^ = 4.39, *P* = 0.036). Two non-toxigenic *C. difficile* isolates in 2014 and 2015, respectively, were not associated with CDI according to the published guideline [23].

**Fig. 2:**
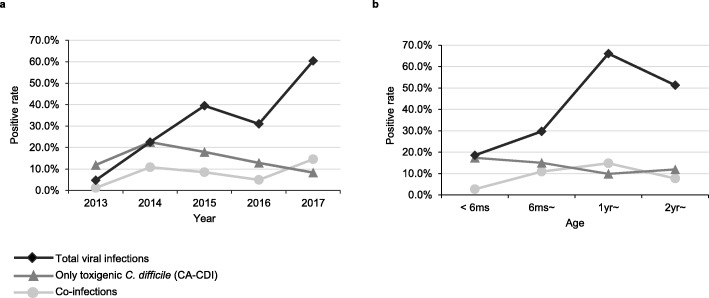
Line chart of the positive rates of only toxigenic *C. difficile* (CA-CDI), total viral infections, and co-infections with different horizontal groups; a: in five years. b: in different age groups

In CA-CDI cases, the positive rates of toxigenic *C. difficile* from 2013 to 2017 were 11.9, 22.5, 17.9, 12.8, and 8.3%, respectively, revealing a significantly declining trend (trend chi-square *χ*^*2*^ = 5.84, *P* = 0.016), while a notable uptrend was observed in the viral infections (trend chi-square *χ*^*2*^ = 73.53, *P* < 0.001), with rates of 4.8, 22.5, 39.5, 31.0, and 60.4% (Fig. 2a). Analysis of the age distribution of pathogens revealed that co-infections were more common in 6 months to 2 years of age (*χ*^*2*^ = 21.38, *P* < 0.001). The positive rates of viral infections were much higher in children aged over 1 year (*χ*^*2*^ = 99.91, *P* < 0.001), while the CDI rates were relatively stable among different age groups (*χ*^*2*^ = 5.22, *P* = 0.156) (Fig. 2b).

## Discussion

Despite a colonization rate of toxigenic or non-toxigenic *C. difficile* of 30–40% in newborns, 30% in infants between 1 and 6 months of age and a reduction to 14% between 6 and 12 months of age, the incidence of CDI has dramatically increased in pediatric populations [34, 35]. Meanwhile, CA-CDI cases have increased in young children [6, 36, 37], along with a simultaneous global increase in studies on CA-CDI in children, in recent years [38–40]. However, studies have been more focused on the colonization of *C. difficile* in children from China [21, 22]. Only one study, on CA-CDI in southwest China, compared the clinical features and molecular characteristics in both children and adults [41]. The prevalence of CA-CDI in young children from China remains unknown.

The positive rate of CA-CDI (14.3%) from this study was consistent with the findings in southwest China (14.3% for children) [41], but higher than that reported in the USA [36, 37, 42] and Europe [43, 44]. According to a meta-analysis, the mean positive rate of toxigenic *C. difficile* in diarrheal adult patients from mainland China was 14% (95% confidence interval = 12–16%) [45]. In our study, the positive rate of CA-CDI showed a downward trend with the increase of viral infections. This was consistent with reports stating that diarrhea in children under 5 years old was mainly caused by viruses, both in China and worldwide [15, 46]. Notably, the positive rate of CA-CDI found in infants was higher than in children over 1 year old in this study, and only two non-toxigenic isolates found were not associated with CA-CDI in children with acute gastroenteritis. Children under 1 year were not recommended to conduct CDI tests due to high rates of *C. difficile* colonization as the new clinical practice guideline described [13]. So, the role of toxigenic *C. difficile* in infants and children has still been controversial in CDI cases. However, there were no any data supporting the standard of CDI diagnosis for children in China. The diversity of individual gut microbiomes was distinctly different among human beings from different geographical regions [47], and stewardship of antimicrobial use was implemented at different time in different counties [48]. Thus, it was speculated that the principle of CDI test for children under 1 year might be differentiated in China. Due to the high positive rate detected in this study, toxigenic *C. difficile* seemed to play an important role in acute gastroenteritis in children from eastern China. Thus, further investigation is required to confirm its role in infants, in order to guide or determine whether clinical CDI tests should be performed.

Our results indicated that the distribution of *C. difficile* genotypes in children from eastern China was distinctly different from those in adult hospitalized patients and in other regions [22, 45, 49]. All the toxigenic *C. difficile* isolates in this study were clustered in clade 1 and 4 in children, with toxigenic ST26 being one of the major genotypes in children mostly under 1 year old in China, and however a molecular epidemiology study in the UK showed that the most common genotypes in children were the non-toxigenic ST26 and ST15 [50]. Furthermore, a systematic review and meta-analysis in mainland China showed that ST2 and ST37 were the dominant genotypes in mainland China [45], and our previous study also identified ST37 as one of the most dominant genotypes associated with sever CDI in hospitalized adult patients from eastern China [25]. However, ST37 was not one of the major genotypes, and accounted for 30.8% of the A^−^B^+^ strains in children in this study, which is similar to the report from southwest China [41]. It was speculated that ST37 might be transmitted into gastrointestinal tract along with individual growing up, which need to be further studied through monitoring continuous changes in intestinal flora. We also found that ST152 rarely reported in adult patients was identified as another major genotype in children. Even though most of the ST152 isolates were obtained in 2015, there were no relationships among these children, including geographical address and daily interaction. Thus, whole genome sequencing should be further performed to investigate the genetic relationships among these *C. difficile* isolates.

High consistencies between antimicrobial related genes and resistance phenotype was found in this study, indicating that the erythromycin- and clindamycin-resistant and the tetracycline-resistant isolates were mainly mediated by *ermB* and *tetM* genes, respectively. The antimicrobial resistance pattern on *C. difficile* isolates presented the low resistance rates to rifampin, moxifloxacin, gatifloxacin and tetracycline, which were similar to those of *C. difficile* isolates from diarrheal adults with healthcare acquired CDI [25, 45]. The antimicrobial resistance data were also compared with those on *C. difficile* isolates from adults with CA-CDI published in our team. The results showed that the CA-CDI associated A^+^B^+^ isolates from children presented significantly higher resistance rate to erythromycin (82.8%, 120/145) than that in adults with CA-CDI (60.8%, 62/102)(*χ*^*2*^ = 14.91, *P* < 0.001), and however A^−^B^+^ isolates in children (87.2%, 34/39) exhibited distinctly lower resistance rate to clindamycin than that from adults with CA-CDI (100.0%, 88/88) (Fisher's exact test, *P* = 0.002) in Zhejiang [51]. It was speculated that frequent and inappropriate antimicrobial usage and different intestinal flora might be main reasons to lead to the differences on antimicrobial resistance in between adults and children in Zhejiang, China, which need be studied in the near future. Notably, we also found that the resistance rates of erythromycin, rifampin, moxifloxacin and gatifloxacin in A^−^B^+^ isolates were significantly higher than in A^+^B^+^ isolates in children, indicating that exposures to clindamycin, erythromycin, fusidic acid, levofloxacin and ciprofloxacin might potentially exist in pediatrics in eastern China. Therefore, antimicrobial resistance mechanisms should be investigated and more antimicrobial resistance genes such as *mefA*, *cfrB*, and *cfrC* should be detected as the previous report [52] later in order to obtain the complete molecular characterization of the *C. difficile* isolates from pediatrics. Furthermore, partial *C. difficile* A^−^B^+^ isolates led to clinical severe CDI as we previously reported [25]. Thus, CDI cases induced by A^−^B^+^ isolates should be treated under the guidance of antimicrobial resistance tests in clinical therapy.

Only two non-toxigenic isolates were found in this study, indicating that acute diarrhea might mainly be induced by toxigenic *C. difficile* in children in Zhejiang, China. The relationship between toxigenic and non-toxigenic isolates was still unclear. Even though no significant differences on the resistance patterns were found between the non-toxigenic and toxigenic isolates except clindamycin, small numbers of non-toxigenic isolates might result in possible in our data analysis results among them. Thus, more non-toxigenic isolates should be collected to explore the correlation of *C. difficile* between with and without toxin genes, and supplement the resistance characteristics of *C. difficile* from children in Zhejiang, China.

There are some limitations in this study. Firstly, only outpatients from one tertiary children’s hospital was enrolled, making selection bias inevitable. Inpatient children should also be involved to disclose the intact molecular characteristics of *C. difficile* in this region. Secondly, a concrete medical record history was unavailable. Clinical information, including history of antibiotic use and clinical diagnosis, should be recorded in order to analyze the risk factors of the increasing prevalence of CDI in diarrheal children. Thirdly, the actual cause of diarrhea in outpatients, co-infected with both viruses and toxigenic *C. difficile,* was still unknown. Thus, we are going to conduct another study with a large scale of diarrheal children from outpatients and inpatients including clinical information and a questionnaire including more risk factors such as environmental exposure with pets [53] in order to analyze molecular epidemiology, transmission routes and risk factors of CDI in children in China, and meanwhile the role of toxigenic *C. difficile* in young children should be investigated later.

## Conclusions

This was the first study on the molecular characteristics of *C. difficile* in outpatient children with acute gastroenteritis, from Zhejiang, eastern China. A wide variety of STs, including ST26, ST54, ST35, ST39, and ST152, were found to be major genotypes with differing antimicrobial resistance profiles in children, which differed distinctly from adults in China. A^−^B^+^ isolates need to be considered due to the high antimicrobial resistance rates. Further studies and surveillance should be performed to investigate the role and risk factors of *C. difficile* in children with diarrhea.

## Data Availability

All data generated and/or analyzed during the current study are available from the corresponding authors on reasonable request.

## Abbreviations

CDI: *Clostridium difficile* infection
CA-CDI: Community-associated *Clostridium difficile* infection
XCCDC: Xiacheng District Center for Disease Control and Prevention
SHEA/IDSA: Society for Healthcare Epidemiology of America and the Infectious Diseases Society of America
CCFA: Cefoxitin-Cycloserine Fructose Agar
ATCC: American Type Culture Collection
MLST: Multi-locus sequence typing
ST: Sequence type
PIP-TAZ: Piperacillin-Tazobactam
CLSI: Clinical and Laboratory Standards Institute
MDR: Multi-drug resistance
IQR: Interquartile range
